# Genetic linkage mapping in *Megathyrsus maximus* (Jacq.) with multiple dosage markers

**DOI:** 10.1093/g3journal/jkaf126

**Published:** 2025-07-16

**Authors:** Gabriel de Siqueira Gesteira, Getulio Caixeta Ferreira, Marcelo Mollinari, Mateus Figueiredo Santos, Liana Jank, Mariane de Mendonça Vilela, Andrea Raposo, Lucimara Chiari, Zhao-Bang Zeng, Antonio Augusto Franco Garcia

**Affiliations:** Bioinformatics Research Center, Department of Horticultural Science, North Carolina State University, Raleigh, NC 27607, USA; Department of Genetics, “Luiz de Queiroz” College of Agriculture, University of Sao Paulo, Piracicaba, SP 13418-900, Brazil; Department of Genetics, “Luiz de Queiroz” College of Agriculture, University of Sao Paulo, Piracicaba, SP 13418-900, Brazil; Bioinformatics Research Center, Department of Horticultural Science, North Carolina State University, Raleigh, NC 27607, USA; Embrapa Beef Cattle, Campo Grande, MS 79106-550, Brazil; Embrapa Beef Cattle, Campo Grande, MS 79106-550, Brazil; Embrapa Beef Cattle, Campo Grande, MS 79106-550, Brazil; Embrapa Beef Cattle, Campo Grande, MS 79106-550, Brazil; Embrapa Beef Cattle, Campo Grande, MS 79106-550, Brazil; Bioinformatics Research Center, Department of Horticultural Science, North Carolina State University, Raleigh, NC 27607, USA; Department of Genetics, “Luiz de Queiroz” College of Agriculture, University of Sao Paulo, Piracicaba, SP 13418-900, Brazil

**Keywords:** linkage map, polyploid, autotetraploid, guineagrass

## Abstract

*Megathyrsus maximus* (Jacq.), commonly known as guinea grass, is a forage crop widely used to form pastures and feed livestock. The species stands out for presenting high yield and nutritional quality in the leaves and its ability to be clonally propagated by seeds. In this work, we construct a dense and informative genetic linkage map for *M. maximus* using multiple dosage markers. We sequenced DNA from leaf samples of 224 individuals from a biparental cross between two tetraploid genotypes, then analyzed the raw sequencing data to find variants and call dosage-based genotypes using four related reference genomes. With the multiple dosage genotypes for both parents and all individuals, we constructed a highly informative genetic linkage map using state-of-the-art methods coupled with the multipoint Hidden Markov Model approach. We present the densest and most informative genetic linkage map to date for the species, with 7,095 markers distributed across eight homology groups, spanning 1573.31 cM of the genome. Both parents and all individuals in the mapping population were phased according to the species’ ploidy level. There was no evidence of double-reduction or preferential pairing in the studied population. The linkage analysis provided in this work can help unravel the evolutionary pathway of the species, understand the genetic behavior of quantitative traits, assist in the assembly of reference genomes, and support the adoption of genomics-assisted selection strategies in *M. maximus* breeding programs.

## Introduction

*Megathyrsus maximus* (Jacq.) B.K. Simon & S.W.L. Jacobs (Syn. *Panicum maximum* Jacq., *Urochloa maxima* (Jacq.) R. Webster), commonly known as guinea grass, is a forage crop widely used in cattle beef production due to its high yield and outstanding nutritional quality. The species originated in East Africa but had great adaptation to different tropical and sub-tropical land areas and became widely cultivated in many countries in South America. In the Brazilian market, it is among the most productive grasses propagated by seeds (Jank *et al.* 2011). The species occurs in two natural forms: sexual diploid (2n=2x=16) and apomictic tetraploid (2n=4x=32) genotypes. Other chromosomal numbers, as well as hexaploids and aneuploids, were also reported in the literature with lower frequencies (Warmke 1951; Jauhar 1969; Savidan 1980; Hamoud *et al.* 1994; Giussani *et al.* 2001; Jain *et al.* 2003; Akiyama *et al.* 2008).

Since natural tetraploid genotypes of *M. maximus* undergo apomixis (i.e. asexual propagation by seeds, Nogler 1984), it is possible to fix superior genotypes and their hybrid vigor while maintaining uniform pastures using genetically identical seeds (Jank *et al.* 2011). Most of *M. maximus* breeding programs have been taking advantage of sexual and apomictic genotypes by combining them in crossing schemes. Thus, sexual genotypes are used to allow recombinations throughout the crosses, while apomixis is used to fix the best genotypes and produce seeds on a large scale through asexual propagation. As sexuality is exclusive to diploid genotypes in nature for *M. maximus*, the first sexual tetraploid genotypes were initially diploids which had their chromosomes duplicated with colchicine to allow viable crossings (Savidan 1980; Nakagawa and Hanna 1992; Nakagawa *et al.* 1993). Previous studies have shown that progenies derived from tetraploid sexual vs. apomictic crosses segregate in a 1:1 rate for apomixis (Savidan 1978, 1981; Ebina *et al.* 2005; Bluma-Marques *et al.* 2014; Deo *et al.* 2020), although other studies suggest a quantitative genetic control for this trait (Kaushal *et al.* 2008, 2019; Marcón *et al.* 2019).

The advancements in molecular technology enabled the detection of variants in DNA and RNA sequences, which allowed the identification of sources of variation on both molecular and phenotypic bases. This knowledge has been supporting studies on DNA recombination, molecular paths, and its interactions, helping to understand the mechanisms that drive phenotypic expression, organism differentiation, and speciation (Metzker 2009; Elshire *et al.* 2011; Poland and Rife 2012). Many of these investigations are based upon analyses on the genetic diversity of a population, linkage and QTL mapping, genomewide association studies, or whole-genome prediction; thus, their outcomes have the potential to significantly change the way breeding programs are planned and conducted (Poland and Rife 2012). Therefore, methods to obtain, evaluate, and analyze molecular datasets are widespread and well-developed, especially for diploid species. However, there has been a delay in developing and extending such technology and methods for polyploid species, mainly due to their genomic complexity and lack of resources (Garcia *et al.* 2013).

The assessment of loci variation for entire populations has become a fundamental part in the development of crop species. Among the available technologies, single nucleotide polymorphism (SNP) is a cost-effective and the most abundant form of variation in the genome, usually in the form of biallelic markers (Brumfield *et al.* 2003). The evaluation of such variants along the genome also allows accessing the allele abundance and the estimation of genotypes in polyploid species (Voorrips *et al.* 2011; Serang *et al.* 2012; Garcia *et al.* 2013; Hackett *et al.* 2013; Gerard *et al.* 2018). Thus, individual genotypes can be represented with different dosages ranging from zero up to the ploidy level of the species. The dosage value usually means the estimated count of the reference allele that an individual carries for a given biallelic locus. As an example, an autotetraploid species may present individual dosages ranging from 0 to 4, which would represent the genotypes aaaa, aaaA, aaAA, aAAA, and AAAA for a biallelic marker (Lara *et al.* 2019). Despite providing more information than single dosage markers (i.e. only nulliplex, simplex, and double-simplex genotype combinations), dosage-based genotypes still lack the complete genetic information for an individual, especially for polyploids. This complete information would include multiple allele information and their distribution across individual haplotypes and along the genome, their phase configurations with adjacent locus, and the origin and recombination events that generated each haplotype in an individual genetic set. Fortunately, state-of-the-art methods can recover the complete genetic information from the same data that generates dosage genotypes, usually by utilizing genomic sequences or performing linkage analysis. The former takes sequence-based information to detect unique haplotype sequences and recover phase configurations or multiallelic information (Motazedi *et al.* 2018, 2019; Moeinzadeh *et al.* 2020; van Geest *et al.* 2020), while the latter uses additional information from the population structure to model the transmission of alleles from parents to the offspring, which include the expected Mendelian segregation rates, linkage phase configurations, and recombination frequencies (Hackett *et al.* 2013; Bourke *et al.* 2018b; Mollinari and Garcia 2019).

Linkage analysis has been widely used to understand genetic conformity and the inheritance pattern in targeted mapping populations. In addition to the identification of linkage groups, the recombination frequencies, physical distances, and the phase configuration between a set of genetic variants, linkage analysis allows to recover the complete genetic information and study the meiotic process involved in the haplotypic inheritance for a given population (Mollinari and Garcia 2019; Mollinari *et al.* 2019). With the complete genetic information, it is also possible to search for QTL along the genome by using the joint genotype probabilities of all individuals (Pereira *et al.* 2020). Only recently, autopolyploid species benefited from the extension of methods to construct integrated genetic linkage maps based on multi-dosage information, primarily for tetraploids (Hackett *et al.* 2013) and hexaploids (Bourke *et al.* 2018b), then extended to take advantage of the Hidden Markov Model to get multilocus estimates for higher ploidy levels (Mollinari and Garcia 2019). The same was observed for QTL mapping models Hackett *et al.* (2013, 2014); Chen *et al.* (2018); Pereira *et al.* (2020). A few polyploid species benefit from high-quality, dense, and integrated linkage maps, while most were constructed based on single-dosage markers (Wu *et al.* 1992) or using diploid-based methods (Balsalobre *et al.* 2017; Shirasawa *et al.* 2017; Ferreira *et al.* 2019). Thus, they lack the informativeness provided by novel sequencing technologies coupled with large populations, good reference genomes, and state-of-the-art statistical methods developed specifically for polyploid organisms.

Several investigations have been conducted to study the *M. maximus* molecular behavior, including genetic diversity studies (Giussani *et al.* 2001; Aliscioni *et al.* 2003; González and Morton 2005; Akiyama *et al.* 2008; Salariato *et al.* 2010; Grass Phylogeny Working Group II 2011; Morrone *et al.* 2012; Hunt *et al.* 2014; Kellogg 2015; Burke *et al.* 2016; Tomaszewska *et al.* 2021), linkage and QTL mapping (Ebina *et al.* 2005; Deo *et al.* 2020), transcriptome and RNAseq analysis (Yamada-Akiyama *et al.* 2009; Toledo-Silva *et al.* 2013; Radhakrishna *et al.* 2018; Wedow *et al.* 2019), genomic selection (Lara *et al.* 2019), and cytogenomics (Tomaszewska *et al.* 2021). However, there is still no consensus regarding the taxonomic placement of the species, and little is known about its evolutionary and genomic behaviors, as well as the molecular pathways that drive phenotypic expression. Although other linkage and QTL studies were reported for *M. maximus*, they lack the density and informativeness provided by recent statistical methods; yet few autopolyploid species have already benefited from them (Ferreira *et al.* 2019; Mollinari *et al.* 2019; Cappai *et al.* 2020; Oloka *et al.* 2021).

Given *M. maximus* relevance to tropical and subtropical livestock farming and the lack of studies involving recent statistical methods, more studies are necessary to unravel the species’ genomic complexity, its inheritance patterns, molecular pathways, and their relation to phenotypic expression. Thus, the objectives of this study were to: (a) detect DNA polymorphisms in a *M. maximus* mapping population; and (b) construct a state-of-the-art genetic linkage map with phased parental and progeny haplotypes. The results of this work will contribute to the advancement of knowledge and support the development of genomic technologies for the species, including the assembly of a reference genome, and also provide a reliable basis for further investigations, such as QTL mapping and genomics-assisted selection, which will help improve the efficiency of *M. maximus* breeding programs.

## Materials and methods

### Mapping population

To obtain the biological samples for this study, we performed a biparental cross between distinct cultivars, namely *Miyagui* and *S12*. The former is an apomictic commercial cultivar used as the male parent (pollen donor), whereas the latter is a sexual accession used as the female parent. We selected these genotypes due to their contrast in important breeding traits, such as forage yield, plant height, inflorescence compactness, and seed shattering.

Before the cross, we performed a clonal propagation of both genotypes in a greenhouse to increase the number of plants while guaranteeing that all female and male plants were genetically identical. We conducted the cross in 2017 at the Embrapa Beef Cattle, Campo Grande, Brazil, following a regular blocking scheme with female clones in the middle and male clones in the borders of the block. The blocking scheme forces male plants to act as a physical barrier to external pollen contamination, whereas female plants are expected to be pollinated only by male pollen.

Assuming no contamination occurred, we collected seeds produced only by the female plants to obtain the progeny individuals. The resulting F1 segregating progeny consisted of 224 individuals used as the basis for the mapping population. Similar to the parents, we clonally propagated all individuals to increase the number of plants, allowing their evaluation throughout replicated trials while guaranteeing their genetic identity.

### Genotypic data

We collected leaf samples from both parents and all progeny individuals to extract their DNA sequences using the QIAGEN DNAeasy Plant kit. The DNA sequences were arranged in 7 plates of 96-plex each, minus two wells for quality control. All progeny individuals were sampled once, while parents were repeated 14 times (two samples per parent by plate). Plates were sequenced at Elshire Group Inc. (Australia) using the genotyping-by-sequencing (GBS) technique. The GBS library was generated following a modified version of the Elshire *et al.* (2011) protocol, with the following changes: 100 ng of genomic DNA, 1.44 ng of total adapters, the genomic DNAs were restricted with the rare-cut PstI enzyme, sequences were marked with combinatorial barcodes, and the library was amplified with 18 PCR cycles. Sequencing was performed using the Illumina HiSeq Xten platform, producing paired-end reads with 150bp for each plate. After trimming both primer and adapter sequences, the resulting variable-length paired-end reads presented three types of combinations: absent, partially, or totally overlapping reads.

With sequence data in hand, we demultiplexed all sample reads and removed their barcodes using the software *axe-demux* (Murray and Borevitz 2018), followed by a quality control analysis using the software *FastQC* (Available at: https://www.bioinformatics.babraham.ac.uk/projects/fastqc/), *FastQ Screen* (Wingett and Andrews 2018), and *MultiQC* (Ewels *et al.* 2016).

Since *M. maximus* does not have a reference genome available, we mapped all sequence reads to six different reference genomes from phylogenetically related species, using the software *Bowtie2* v2.1.0 (Langmead and Salzberg 2012). All runs included the flag *–very-sensitive-local* to perform a restrictive alignment, limiting the number of dynamic programming problems to 20, the maximum number of alignments of each read to 3, and the size of seeds to 20 bases. The six reference genomes used along the pipeline were: *Panicum hallii* v3.0 (Lovell *et al.* 2018), *Panicum virgatum* v5.0 (Lovell *et al.* 2021), *Setaria italica* v2.0 (Bennetzen *et al.* 2012), *Setaria viridis* v2.0 (Mamidi *et al.* 2020), and *Urochloa ruziziensis* v1.0 (Pessoa Filho *et al.* 2019). All species used as references are diploids with 2n=2x=18 and reference genomes arranged in 9 chromosomes, except the allotetraploid *P. virgatum* (2n=4x=36) with a reference genome arranged in two sets of 9 chromosomes (named as K and N subgenomes). In this case, we mapped the sequence reads to the two subgenomes separately. All published genomes were downloaded from the *Phytozome* v13 database (Goodstein *et al.* 2011) or the NCBI platform (https://www.ncbi.nlm.nih.gov/).

**Fig. 1.**
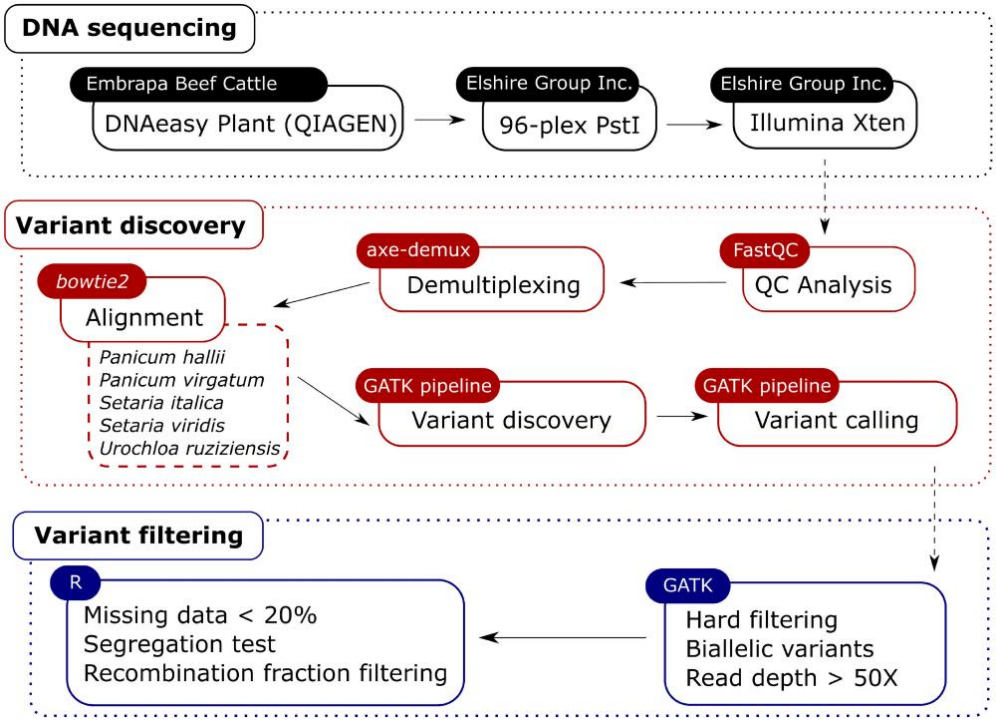
Workflow of the variant discovery and genotype calling pipeline.

With the mapped reads in hand, we performed the variant discovery using the software *GATK* v.4.1.6.0 (McKenna *et al.* 2010), and jointly genotyped all samples simultaneously according to the Best Practices recommendations (submodules: HaplotypeCaller, GenomicsDBImport, GenotypeGVCFs, VariantFiltration, and SelectVariants, Van der Auwera and O’Connor 2020) with the following modifications: no duplicates were removed, and no base or variant recalibration was performed. We also hard-filtered variants according to the Best Practices guidelines by visualizing each parameter distribution separately. We considered the following parameters in the filtering step: total read depth (DP), mapping quality (MQ), mapping quality rank-sum (MQRankSum), quality by depth (QD), fisher strand (FS), and strand odds ratio (SOR). We performed additional filtering steps to consider only biallelic variants with an average read depth higher than 50×. An overview of all steps in the variant and genotype calling steps is shown in Fig. 1.

Finally, we evaluated the fit of all progeny individuals to the biparental cross by their estimated genetic distances to the parents, using the genomic relationship matrix (*G*) (VanRaden 2008). We calculated the *G* matrix using the *R* package *AGHmatrix* v2.0.0 (Amadeu *et al.* 2023) and plotted the genetic distances using the software R (R Core Team 2023).

### Genetic linkage mapping

Before constructing the genetic linkage map, we submitted variants to another filtering round by selecting them according to their informativeness (i.e. non-monomorphic markers with dosage information for both parents) and removing markers with >20% of missing data. Individual genotypes supported by <50 reads were considered missing data as well. We then tested variants using a chi-squared distribution based on the expected Mendelian segregation ratio given each parental dosage combination. The threshold for accepting the null hypothesis (i.e. assuming that a variant follows the expected segregation distribution) was defined using the Bonferroni’s correction (Bonferroni 1936). After removing distorted variants, we identified and temporarily removed redundant variants (i.e. variants that carry the same information), keeping only the first variant of each redundancy group. We temporarily removed those markers because they do not provide additional information to the model (i.e. all variants in the redundancy group would end up in the same map position), but would increase the usage of resources and computation time.

Given the variants that passed through all filtering steps, we estimated the recombination fractions for all possible linkage phases between all pairs of variants, according to Mollinari and Garcia (2019). We used a heatmap graphic to identify linkage evidence between genomic sequences, then manually assigned linked genomic sequences to the same homology groups. We applied another round of filtering by removing any variants with >90% of recombination fractions <0.05 or >0.40 with the remaining markers in their respective homology group. Then, we converted all recombination fractions to genetic distances using Haldane’s mapping function and ordered all variants inside each homology group using the multidimensional scaling (MDS) algorithm (Preedy and Hackett 2016).

Considering the MDS-based variant order for each homology group, we re-estimated their linkage phase configurations and recombination fractions using the multipoint, Hidden Markov Model (HMM) approach extended to autopolyploids by Mollinari and Garcia (2019). We estimated parental linkage phases by sequentially adding variants to the map and evaluating all possible phase configurations between the inserted variant and the existing map sequence. Phase configurations with a difference of <50 on the LOD scale were retained to be evaluated during the next rounds of variant insertion. Similarly, recombination fractions with a difference of <10 on the LOD scale were also retained. The HMM likelihood was calculated for each round using a tail sequence of 200 variants. Markers that inflated the map by >5 cM were removed during the process. All recombination fractions were re-estimated using the full HMM after adding the last variant to the map. With the final maps in hand, we re-estimated all distances considering a global error rate of 5% in the emission function of the HMM. Later, we reinserted the redundant variants at their respective positions and generated the final map for each homology group.

To assess the pairing pattern among haplotypes during the meiosis, we marginalized their probabilities conditional on parental linkage phases and the recombination fractions at each map position, according to Mollinari *et al.* (2019). We also calculated the Genomic Information Content (GIC) among parental haplotypes in the final genetic linkage maps (Bourke *et al.* 2018a). All genetic linkage analysis were performed using the *R* package *MAPpoly* v0.2.3. We developed a web application using the *R* package *shiny* v1.6 (Chang *et al.* 2021) with interactive versions of the final genetic linkage maps and the estimated parental haplotypes The application is available at https://statgen.esalq.usp.br/megathyrsus-map/.

## Results

### Library preparation and genotype-by-sequencing

The GBS library amplified well and presented a good fragment size distribution. Sequencing was also performed successfully, providing high-quality paired-end reads for all plates. The library presented an average of 437.57 million paired-end reads and particularly outstanding coefficients of variation, 28.28% on average (Table 1), which is far below the averages reported by similar studies (e.g. 43% in maize (Elshire *et al.* 2011), 67% in *D. simulans* (Andolfatto *et al.* 2011), and 39% in beef cattle (Donato *et al.* 2013)). On average, 94.62% of the processed reads were successfully demultiplexed, and all blank checks passed the tests accordingly. The Elshire Group Inc. reported that the library exceeded most of its quality control metrics. One sample in Plate 3 presented a low average of reads due to low DNA concentration, but the aforementioned sample does not belong to the mapping population.

**Table 1.** Overall quality control metrics regarding the GBS library sequencing for all plates.

Plate	Yield (ng/μl)	TRP (Million)	ARPC (Million)	CV (%)	BC	SBA
1	24.3	443	4.4	23	PASS	0
2	28.2	424	4.2	21	PASS	0
3	28.6	449	4.5	25	PASS	1
4	31.4	442	4.4	29	PASS	0
5	27.3	437	4.5	39	PASS	0
6	34.0	442	4.5	26	PASS	0
7	24.4	426	4.4	35	PASS	0

TRP, total read pairs; ARPC, average read pair count; CV, coefficient of variation; BC, blank check; SBA, samples with <10% average.

Our quality control analysis confirmed that the sequencing delivered high-quality reads for all samples in the mapping population. Supplementary Fig. 1 shows that all reads exhibited high mean quality scores for all base pairs along the sequences. The first 110 base pairs presented quality mean scores >35, while the following 20 base pairs stayed >28. Even the last few bases presented scores >25 on the phred-scale for all samples in the mapping population. Similarly, Supplementary Fig. 2 shows that most sequences had a high overall quality score >28, while most remained >38 on the phred-scale.

All samples passed additional quality control tests, including sequence count distribution, per base sequence content, per sequence GC content, per base N content, sequence length distribution, sequence duplication levels, overrepresented sequences, and adapter content. Only the sequence duplication levels produced warnings because >90% of the sequences were duplicated, a behavior that is naturally expected for RAD-seq datasets (Van der Auwera and O’Connor 2020).

### Variant discovery and genotype calling

All reference genomes delivered intermediate levels of mapped reads and number of variants (Table 2). The *U. ruziziensis* reference outperformed the other reference genomes, presenting the highest values for all metrics: 49.84% of mapped reads and 866,361 variants, yielding 12,549 variants after the quality-control filtering steps . On the other hand, the *P. virgatum* subgenomes K and N provided the lowest values: 26.83% and 26.54% of mapped reads, 550,295 and 542,114 variants, yielding 7,843 and 7,983 variants after the quality-control filtering step, respectively. The *P. virgatum* subgenomes also produced the highest level of missing data and the lowest amount of redundancy.

**Table 2.** Percentage of mapped reads, number of initial variants, number of variants AQCF (after quality-control filtering), percentage of missing data, and redundancy rate, for all reference genomes.

Genome	Mapped reads (%)	Variants	Variants (AQCF)	Missing data (%)	Redundancy (%)
*U. ruziziensis*	49.84	866,361	12,549	20.35	10.31
*P. virgatum K*	26.83	550,295	7,843	21.74	8.95
*P. virgatum N*	26.54	542,114	7,983	22.30	9.18
*P. hallii*	31.91	671,353	9,108	19.74	10.26
*S. italica*	36.18	765,041	10,314	19.88	10.08
*S. viridis*	36.23	770,317	10,683	20.12	9.85
Total	36.91	5131,997	79,534	20.74	9.98

Annotated gene information for all genomes except the *U. ruziziensis*, is available at the *Phytozome* v13 platform (Goodstein *et al.* 2011). The pairwise orthology plots between the available genomes show that relatively high collinearity exists for both gene content and position along the genomes (Supplementary Figs. 36–41). The annotated genes in the *P. hallii* genome share a very similar order with their correspondents in the *P. virgatum* genome, highlighting the collinearity between the two subgenomes in the *P. virgatum* genome. However, the curve is not perfectly linear, meaning chromosome segments may present different physical lengths and local arrangements between the two genomes. The opposite is observed between *S. italica* and *S. viridis* genomes, as they show a perfect linear relation between their gene positions. The previous pattern can be confirmed as *P. virgatum* presents high collinearity with both *S. italica* and *S. viridis*, despite two small inversions at the beginning of chromosomes 1 and 5. Similarly, the linear relation between *S. italica* and *S. viridis* is confirmed through the identical patterns between the *P. hallii* and both *S. italica* and *S. viridis* genomes. Two whole-sequence inversions are shown in chromosomes 4 and 5, followed by small inversions at the beginning of chromosomes 1 and 5 and in the middle of chromosomes 3 and 4. Despite the high collinearity evidenced by the orthology plots, only 863 variants out of 42,654 in the dataset were redundant between different reference genomes, whereas 4,806 were redundant within reference genomes.

Both markers and individuals presented varying levels of missing data, mostly below the 20% line. Individuals 138, 152, and 123 presented the highest percentages of missing data, 27.64%, 27.22%, and 26.23%, respectively, while the remaining ones stayed in the range between 17.5% and 22.5% (Supplementary Figs. 3 and 4). After removing markers with >20% of missing data, the amount of missing data for individuals was reduced considerably (Supplementary Fig. 5). The remaining percentages of individual missing data passed the threshold level of 20%, thus no individuals were removed due to high missing data levels.

### Genetic linkage mapping

A preliminary analysis of the estimated pairwise recombination fractions between all markers inside each genome provided a better understanding of the fit between the sequence data and the available reference genomes. All genomes produced the expected pattern of a mapping population, with a gradient of hot (red) colors between markers from the same chromosome and cold (blue) colors between markers from different chromosomes (Supplementary Figs. 7–12). Also, there is evidence of linkage between two chromosomes within each genome, thus reducing the number of homology groups to eight and meeting the basic chromosomal number for the *M. maximus* species. Despite showing the expected pattern for a mapping population, it was difficult to include markers yielded by the *P. virgatum* references due to an increased amount of noise and reduced data quality. Thus, we discarded these markers and continued the analysis with markers yielded by the remaining reference genomes.

The variant discovery process provided a total of 79,534 variants distributed in four reference genomes. Some variants presented redundancy, missing genotype information for one or both parents, as well as non-informative dosage combinations, i.e. dosage 0 or 4 for both parents. Those redundant, missing, and non-informative markers were removed, reducing the dataset to 42,654 variants, characterized by 20% of overall missing data, 10.13% of redundancy, and varying dosage combinations between parents (Table 3). The dataset was then filtered again to hold only 20% of missing data on the variant basis, which yielded 28,827 variants with a similar proportion of dosage combinations (Supplementary Fig. 13).

**Table 3.** Dosage combinations between parents Miyagui and S12, and their respective number of variants along some filtering steps used to construct the genetic linkage map.

Miyagui	S12	Variants	Variants (NVM 20%)	Variants (AST)
0	1	7,040	5,470	2,716
0	2	2,855	1,949	253
0	3	1,294	45	34
1	0	9,189	7,203	3,848
1	1	1,511	1,202	529
1	2	848	658	161
1	3	455	160	92
1	4	763	21	18
2	0	3,677	2,509	196
2	1	733	581	153
2	2	919	852	150
2	3	713	574	138
2	4	1,758	1,207	128
3	0	1,529	66	53
3	1	350	123	86
3	2	623	471	133
3	3	1,201	960	546
3	4	3,773	2,816	1,812
4	1	482	17	13
4	2	963	618	81
4	3	1,978	1,325	678
Total		42,654	28,827	11,818

NVM, number of variants after filtering for 20% of missing data; AST, number of variants after the segregation test.

Most markers in the final dataset presented single dosage combinations (0-1, 1-0, 3-4, and 4-3), followed by duplex combinations (2-0, 0-2, 2-4, and 4-2) and multiplex combinations. The amount of missing data was randomly distributed across markers and individuals. A fraction of 12.2% of the markers was removed during the map building process due to redundancy, then added back to the final linkage maps later (Supplementary Fig. 6).

In addition to the marker filtering steps, we used the aforementioned dataset to calculate the genomic matrix (G) and evaluate the genetic distance between all offspring individuals and their parents. Nine individuals in the offspring presented skewed genetic distances to parents when compared to the expected pattern for a biparental cross, of which five exhibited accentuated skew towards the apomictic parental (Supplementary Fig. 16). Because those skewed individuals showed unexpected behavior for a biparental population, we assumed they were contaminants and removed them from the final dataset.

Given the filtered dataset, we performed the chi-square segregation test considering the *P*-value threshold for significance equal to $1.734485\times 10^{-6}$, according to Bonferroni’s correction. This step eliminated 59% of the variants (Supplementary Fig. 14), reducing the number of variants in the dataset to 11,818 (Supplementary Fig. 15).

With the final dataset in hand, we estimated the pairwise recombination fractions between all markers, considering all reference genomes together. As pointed out in the preliminary analysis, there is evidence of linkage between chromosomes within genomes, as well as between chromosomes across genomes, indicating that the inheritance pattern was captured successfully throughout the analysis. Thus, we rearranged the chromosomes according to their linkage patterns and got exactly eight homology groups, which is the basic chromosomal number of the *M. maximus* species and the expected number of homology groups (Supplementary Figs. 17 and 18). Thus, instead of applying any grouping algorithm, we used the available genomic information to group variants according to their linkage evidence.

With the formed homology groups and their respective recombination fractions, we filtered which phase configurations and recombination fractions would be used during the ordering step. Basically, we defined LOD-score thresholds of 5 and 10 for phases and recombination fractions, respectively, to be selected among all possible phases for a given pair of markers. This means that any phase or recombination fraction with LOD scores lower than the thresholds was also considered during the ordering step. We also eliminated variants with >90% of estimated recombination frequencies <0.05 or >0.45 across their respective homology groups (i.e. completely linked or unlinked markers). Finally, we ordered all variants within groups using the Multidimensional Scaling (MDS) algorithm.

**Fig. 2.**
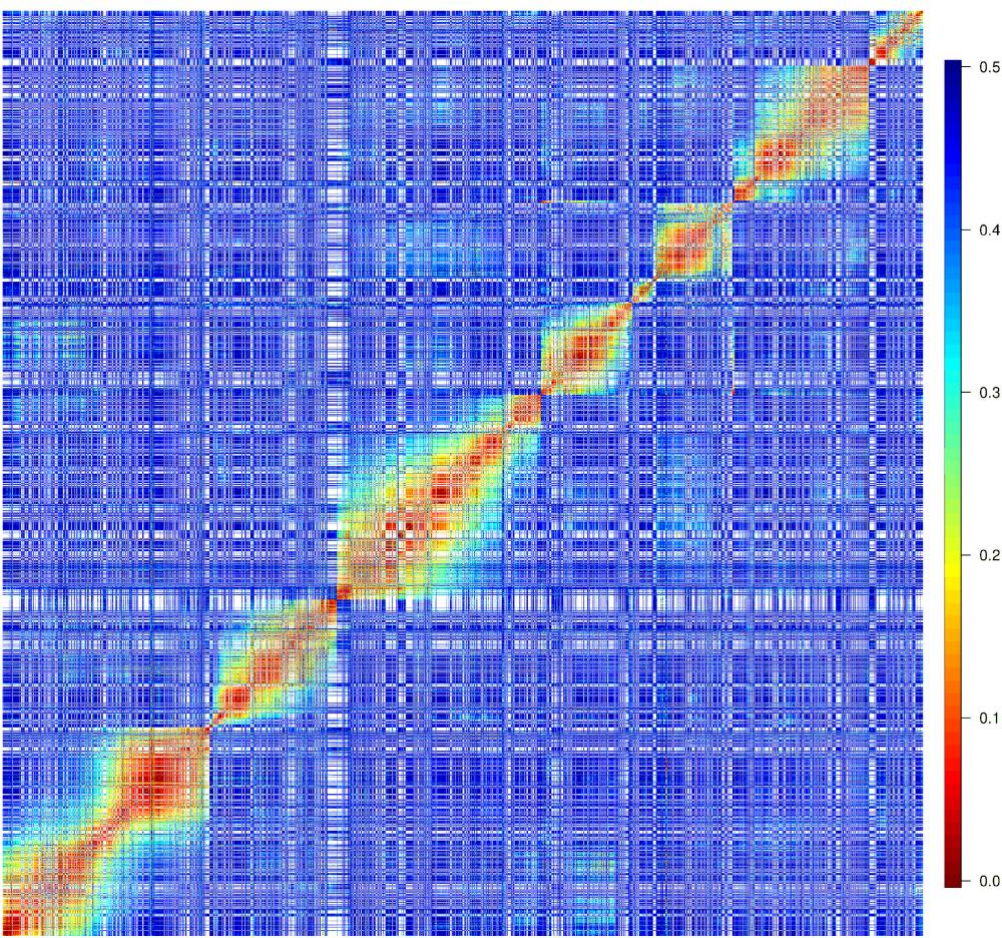
Heatmap of the estimated recombination fractions between markers produced by aligning the sequence data to the following reference genomes: *U. ruziziensis*, *P. hallii*, *S. italica*, and *S. viridis*. Chromosomes were rearranged among genomes to match linkage evidence.

In the final heatmap, a good linkage pattern stands through a gradient of hot (red) colors between variants from the same homology group, while pure blue is shown between variants in different homology groups (Fig. 2). Regarding the number of markers, homology groups 1 and 3 were the biggest ones, followed by homology groups 7, 2, 4, 6, and 8. Homology group 5 was the smallest one, with <200 markers.

**Fig. 3.**
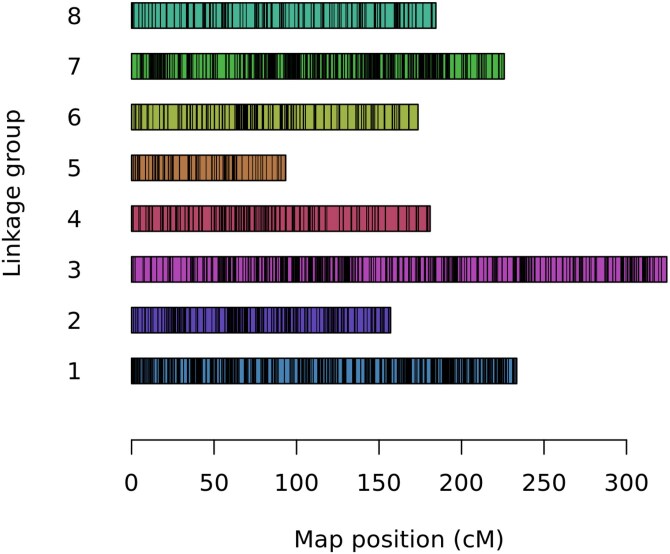
Distribution of markers and their estimated genetic positions (in cM) within each homology group. Colored bars represent different homology groups, while black lines within colored bars represent marker positions.

Based on the estimated order for each homology group, we constructed the maps using the sequential algorithm coupled with the Hidden Markov Model (HMM) and considering a 5% of error rate in the HMM’s emission function. Homology group 3 was the biggest one with 324.41 cM, followed by homology groups 1 and 7 with 233.48 and 225.9 cM, respectively. The homology groups 8, 4, 6, and 2 presented sizes between 184.46 and 157.01 cM, while homology group 5 was the smallest, with 93.43 cM (Fig. 3). Homology group 1 was the densest, with 7.65 markers/cM, while homology group 5, with 1.22 markers/cM was the less dense. The other homology groups presented densities between 2.26 and 5.23 markers/cM. The greatest distance between a pair of markers was 7.55 cM in homology group 4. All homology groups were predominantly composed of simplex markers, with few double-simplex and multiplex variants (Table 4). Also, the final linkage maps were mostly supported by markers produced by the alignment with *U. ruziziensis* reference genome, with 2,134 markers, followed by *S. viridis*, *S. italica*, and *P. hallii* with 1,765, 1,713, and 1,483 markers, respectively (Table 5). The quality of all homology groups increased after removing the previously identified skewed individuals (Supplementary Table 1).

**Table 4.** Overview of the final linkage maps, including their respective sizes, densities, number of markers for each category, and maximum gap size.

HG	Genomic sequence	Size (cM)	Markers/cM	Simplex	D-simplex	Multiplex	Total	Max. gap
1	UR^1^,PH^9^,SI^9^,SV^9^	233.48	7.65	1,527	55	20	1,786	4.14
2	UR^2^,PH^2^,SI^2^,SV^2^	157.01	5.22	728	7	12	820	3.87
3	UR^3,9^,PH^1,4f^,SI,^1,4^,SV^1,4^	324.41	5.23	1,456	47	22	1,698	4.98
4	UR^4^,PH^3^,SI^3^,SV^3^	180.98	3.23	515	11	8	585	7.55
5	UR^5^,PH^8^,SI^8^,SV^8^	93.43	1.22	103	0	1	114	4.98
6	UR^6^,PH^7^,SI^7^,SV^7^	173.64	3.24	484	5	15	563	6.25
7	UR^7^,PH^5^,SI^5^,SV^5^	225.9	4.93	952	8	36	1,113	4.99
8	UR^8^,PH^6^,SI^6^,SV^6^	184.46	2.26	349	23	14	416	5.6
Total		1,573.31	4.12	6,114	156	128	7,095	5.3

HG, homology group. Genomic sequences: UR, *U. ruziziensis*; PH, *P. hallii*; SI, *S. italica*; SV, *S. viridis*. Superscripts represent contigs.

**Table 5.** Number of markers in the final linkage maps by reference genome. All referenced species are diploids with 2n=2x=18 chromosomes.

HG	*S. viridis*	*P. hallii*	*S. italica*	*U. ruziziensis*	Total
1	431	394	417	544	1,786
2	201	147	204	268	820
3	436	367	428	467	1,698
4	132	131	131	191	585
5	33	14	30	37	114
6	140	124	127	172	563
7	278	245	260	330	1,113
8	114	61	116	125	416
Total	1,765	1,483	1,713	2,134	7,095

HG, homology group.

**Fig. 4.**
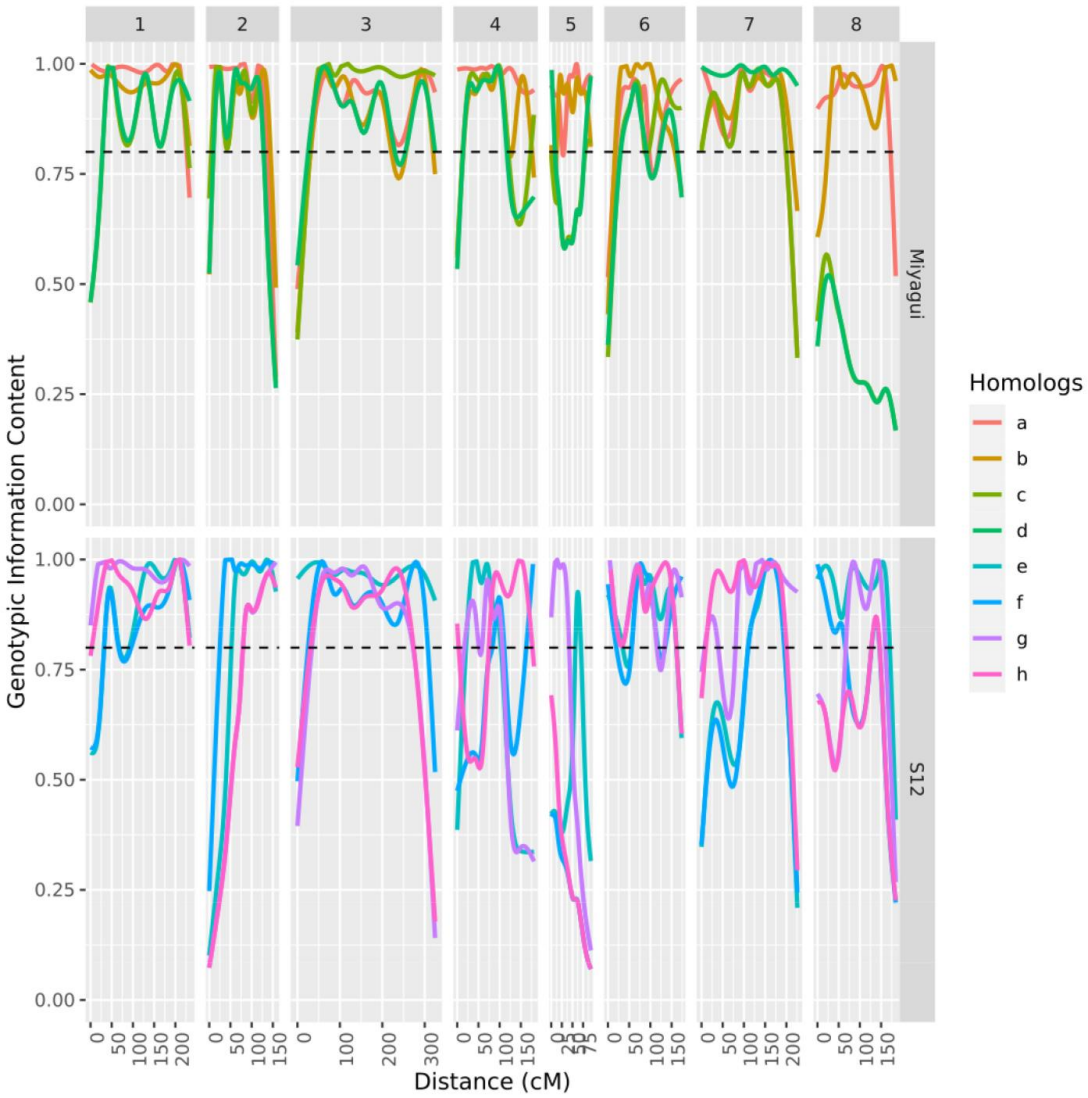
GIC for all haplotypes in both parents.

The GIC graphic shows the amount of information each haplotype carries, allowing their distinction during the map building process. Notably, almost all haplotypes were distinguished well, mostly because of the propagation of information through the chain enabled by the HMM model. However, some haplotypes presented lower levels of GIC because they presented almost identical haplotypes, which hindered their distinction under the assumed threshold levels in the HMM. It is important to note that regions with lower GIC values tend to present very similar haplotype compositions within parents (Fig. 4 and Supplementary Figs. 19–26).

Finally, we performed an evaluation of the pairing configurations and their associated probabilities, given the estimated genotypes for all individuals in the population. All pairing configuration probabilities were close to the 0.33 expected ratio, suggesting no preferential pairing occurred during the meiotic processes that generated this population. The associated *P*-values of all pairing configurations were below the 2.0 threshold, which reinforces that they occurred randomly, following the expected behavior for an autopolyploid species (Supplementary Fig. 27).

## Discussion

The present study reports the third linkage map of *M. maximus* published to date. The high-resolution map represents 64 parental homologs distributed across eight homology groups, which were densely saturated with variants supported by four reference genomes of phylogenetically related species. The 7,095 markers in the final linkage map covered 1,573.31 cM with an average density of 4.12 markers/cM. The first linkage map of the species was released by Ebina *et al.* (2005), which used 360 AFLP and RAPD markers to map 39 linkage groups in a population of 71 individuals, covering 1,703.5 cM for only one parent with an average density of 0.21 marker/cM. Then, a second linkage map was constructed by Deo *et al.* (2020) using the NGS (next-generation sequencing) technology to generate sequence data for both parents and 132 offspring individuals. The authors provided a more informative consensus linkage map, with 858 markers distributed across eight homology groups, totaling 756.69 cM with an average density of 1.13 markers/cM. Thus, the present linkage map provides a great advance and knowledge regarding the species’ genomic conformity through higher resolution, density, and informativity.

Many factors may have contributed to the advance over the previously published linkage maps, including the population size, the choice of the GBS protocol, the use of different reference genomes to discover variants, and the statistical genetics methods employed to build the linkage map. The present linkage map was built upon a larger offspring size of 223 individuals, which gives more statistical power and reduces bias and errors when estimating parameters that rely on sample size, such as linkage phases and recombination fractions. These parameters can influence the genetic linkage analysis in a cascade effect, where linkage phases and recombination fractions influence each other in a two-way fashion, which affect marker grouping and ordering, while these ultimately impact the resulting genetic linkage maps. We highlight that the methods used hereby provided a complete framework to build the map with all the information available, using all possible dosage combinations in an integrated and multilocus-based approach (Mollinari and Garcia 2019). This framework can overcome possible limitations imposed by reduced sample size or a small number of variants, mostly because of the propagation of information enabled by the transitive property of the multipoint HMM approach. Thus, all phases and recombination fractions are jointly estimated based on the information present in the whole homology group, rather than solely on the pairwise estimates. Furthermore, this allowed the inference on the transmission of parental haplotypes to the offspring through haplotype probabilities, enabling the reconstruction of all individual haplotypes in the population (Mollinari *et al.* 2019).

The relatively low number of variants provided by the *P. virgatum* genome coupled with its inability to detect the linkage pattern in this study, even considered its collinearity with the other genomes such as *P. hallii* (Lovell *et al.* 2018) and *S. italica* (Daverdin *et al.* 2014), drew our attention to the possible reasons for this outcome. Deo *et al.* (2020) chose the *P. virgatum* genome as a reference due to its phylogenetic proximity to *M. maximus* (Burke *et al.* 2016), highlighting that the species used belong to the Panicum genus. The authors also emphasize that *P. virgatum* has an allotetraploid genome, and using it as a reference would provide more information than a diploid genome, as it might share similar chromosomal rearrangements with the autotetraploid genome (Deo *et al.* 2020). On the other hand, Burke *et al.* (2016) found out that *M. maximus* was phylogenetically closer to *S. italica* than *P. virgatum*, which also has a reference genome available. As highlighted by Deo *et al.* (2020), the species used to belong to the Panicum genus (subgenus Megathyrsus, Pilger 1931; Zuloaga 1987), but further investigation supported its placement under the Urochloa genus due to morphological, biochemical, and genomic evidences (Webster 1987; Giussani *et al.* 2001; Aliscioni *et al.* 2003; Masters *et al.* 2024). Despite changes in its taxonomic placement, it remains evident that *M. maximus* is phylogenetically closer to Urochloa, followed by Setaria and Panicum species (Webster 1987; Zuloaga 1987; Duvall *et al.* 2001; Giussani *et al.* 2001; Aliscioni *et al.* 2003; González and Morton 2005; Salariato *et al.* 2010; Grass Phylogeny Working Group II 2011; Morrone *et al.* 2012; Hunt *et al.* 2014; Kellogg 2015; Burke *et al.* 2016; Tomaszewska *et al.* 2021). This could explain the better performance of the *U. ruziziensis* genome to yield variants when compared with references from other genera, such as Setaria and Panicum.

The number of homology groups identified in the present study agrees with the basic chromosomal number of the species, which was previously reported by several investigations (Warmke 1951; Jauhar 1969; Savidan 1980; Hamoud *et al.* 1994; Jain *et al.* 2003; Akiyama *et al.* 2008). Interestingly, two chromosomes from all reference genomes supported the same homology group in the *M. maximus* mapping population, with a strong and evident linkage pattern across all markers in these chromosomes. Moreover, the linkage pattern suggests that this homology group is formed by two pieces of a segmented chromosome located at its ends, with the other chromosome inserted in the middle of the homology group, between these two pieces, a pattern evidenced by all reference genomes. We speculate that this pattern may be related to a chromosomal rearrangement that might have played an important role in the evolutionary path of *M. maximus* and its related species, especially the ones with basic chromosomal numbers equal to eight and nine. To the best of our knowledge, the present study reports the first evidence of a chromosomal rearrangement in the evolutionary path of *M. maximus* and related species.

Most homology groups presented a relatively good collinearity between the physical orders from the reference genomes and the global order of the linkage maps. It is also possible to identify plateaus where no recombinations were observed near the center of the physical vs. map position plots (Supplementary Figs. 28–35). These regions are likely associated with centromeres, which suggests that the chromosomes are predominantly metacentric and submetacentric, and agrees with previous reports in the literature for the species (Hamoud *et al.* 1994; Akiyama *et al.* 2008). There is an apparent map inflation for almost all homology groups that could be related to a local misplacement of closely linked markers, as well as the use of several markers produced by different reference genomes, which may have led to an accumulation of small genotyping errors. Almost all homology groups presented sizes between 157.01 and 233.48 cM, which also agrees with previous studies with similarly sized chromosomes in *M. maximus* (Hamoud *et al.* 1994), except for homology groups 3 and 5. As previously discussed, homology group 3 may be inflated due to the presence of 1,698 markers from eight reference sequences, possibly in locally misplaced positions. For homology group 5, we speculate that it might be underrepresented in this study because: (1) a high portion of the sequence data (50% of sequence reads) was not mapped to any region in the reference genomes, and as such, not utilized in this study; (2) a very low number of markers supported it; (3) there is an absence of collinearity between the linkage map and the physical orders of markers in the reference genomes utilized. Thus, we speculate that although genomic regions from this chromosome (hereby represented by homology group 5) might have been properly sequenced in our study, the absence of similar regions in the reference genomes utilized could have led to a lower representation of sequence reads coming from this specific chromosome, which also suggests its uniqueness to the *M. maximus* genome.

The present study reports for the first time the complete set of haplotypes for parents of a population in *M. maximus*. Almost all homologs inside each parent were fully distinguished from each other by using the information contained in all dosage markers. There was only one pair of almost identical homologs inside each parent: homologs c and d from homology group 8 for parent Miyagui (Supplementary Fig. 26); and homologs f and h from homology group 5 for parent S12 (Supplementary Fig. 23). The high similarity between these homologs was captured through the GIC plot (Fig. 4), where GIC values were close to or <0.5, and represents the inability of the markers to capture distinct regions between homologs within parents. Specifically for parent S12, this could be related to the speculated underrepresentation of homology group 5, which was supported by only 114 markers. On the other hand, all homologs from both parents in the remaining homology groups presented high GIC values, which reflects their ability to carry enough information to distinguish homologs along the linkage maps. We also assessed the probability of each possible pairing configuration between parental homologs during meiosis. Supplementary Fig. 27 shows that there was no evidence of preferential pairing in the formation of the mapping population, suggesting that pairing occurs randomly and mostly in the bivalent form, which reinforces the autopolyploid nature of the *M. maximus* genome. This result agrees with previous reports in the literature regarding the predominant autopolyploid nature of the genome, the absence of preferential pairing, and the predominant occurrence of random bivalent pairing during meiosis in *M. maximus* (Warmke 1951; Jauhar 1969; Jain *et al.* 2003).

Having fully estimated haplotypes for parents and individuals in a population, such as the ones generated in this study, provides a much more informative framework for further genetic studies, such as QTL mapping, and enable their use in several downstram applications, including genomics-assisted selection and genomic prediction. The highly informative genetic linkage map generated hereby can also serve as a basis for the assembly of a reference genome for the species, where fully-phased haplotypes can be especially helpful to resolve the multiple challenges involved in autopolyploid, highly heterozygous genomes such as *M. maximus*. The genetic knowledge presented hereby, together with all possibilities that are enabled with it, can help pave the path towards the adoption of novel technologies and allow for genomics-informed decisions that can help increase the efficiency of *M. maximus* breeding programs.

### Conclusion

We were able to detect DNA variants and map them to the densest and most informative linkage map of *M. maximus* up to date. We also provided phased haplotypes for all individuals in the mapping population and studied the type of genetic inheritance in *M. maximus*.

The present investigation provides new insights into the genomic behavior and evolutionary pathway of *M. maximus*, producing more evidence for its evolutionary placement among other relative grasses, and providing more support to the latest taxonomic classification of the species. Thus, new speculations can be drawn over the genomic origin and relatedness between *M. maximus* and other related species, helping to solve the puzzle of the relationship between the basic chromosomal number and the evolutionary pathways between Panicoidae species.

The linkage map can help to assemble a reference genome for the species, thus encouraging and providing better information for future studies. This study also provides the basis for further investigation that can help unravel the genetic behavior of complex traits, including QTL mapping, and support the development of a framework to support breeding programs of *M. maximus*, which include marker-assisted selection and genomic prediction.

## Data Availability

The raw genotype data of the entire mapping population is divided into five VCF files, one for each reference genome utilized, and made available as Supplementary Material at figshare: https://doi.org/10.25387/g3.27208329. The final genetic linkage maps and estimated parental haplotypes can be accessed through an interactive *Shiny* app available at: https://statgen.esalq.usp.br/megathyrsus-map/.
